# Bioprocess microfluidics: applying microfluidic devices for bioprocessing

**DOI:** 10.1016/j.coche.2017.09.004

**Published:** 2017-11

**Authors:** Marco PC Marques, Nicolas Szita

**Affiliations:** Department of Biochemical Engineering, University College London, Bernard Katz Building, Gordon Street, London WC1H 0AH, United Kingdom

## Abstract

Scale-down approaches have long been applied in bioprocessing to resolve scale-up problems. Miniaturized bioreactors have thrived as a tool to obtain process relevant data during early-stage process development. Microfluidic devices are an attractive alternative in bioprocessing development due to the high degree of control over process variables afforded by the laminar flow, and the possibility to reduce time and cost factors. Data quality obtained with these devices is high when integrated with sensing technology and is invaluable for scale-translation and to assess the economical viability of bioprocesses. Microfluidic devices as upstream process development tools have been developed in the area of small molecules, therapeutic proteins, and cellular therapies. More recently, they have also been applied to mimic downstream unit operations.

## Highlights

• Microfluidic devices as novel bioprocess development tools.
• Processes with stem cells, microbes and enzymes are viable in microfluidic devices.
• Microfluidic devices with integrated sensors provide high quality data.
• Laminar flow enables spatial and temporal control over transport phenomena.
• Standardization of devices required for automation and industrial uptake.

## Graphical abstract

**Figure:**
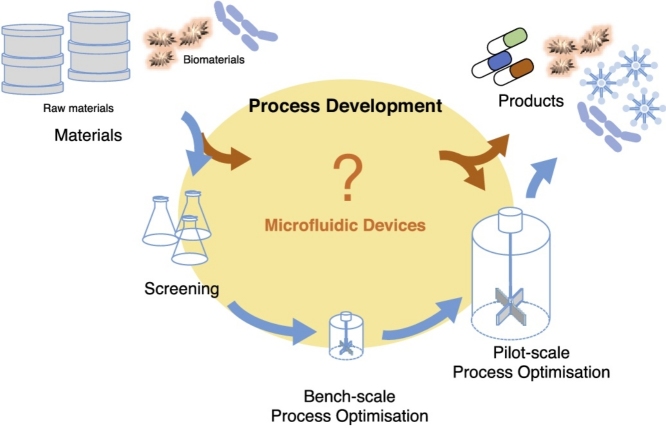

**Current Opinion in Chemical Engineering** 2017, **18**:61–68

This review comes from a themed issue on **Biotechnology and Bioprocess Engineering**

Edited by **Nigel J Titchener-Hooker**

For a complete overview see the Issue and the Editorial

Available online 10th November 2017

**https://doi.org/10.1016/j.coche.2017.09.004**

2211-3398/© 2017 Published by Elsevier Ltd.

## Introduction

**Figure 1:**
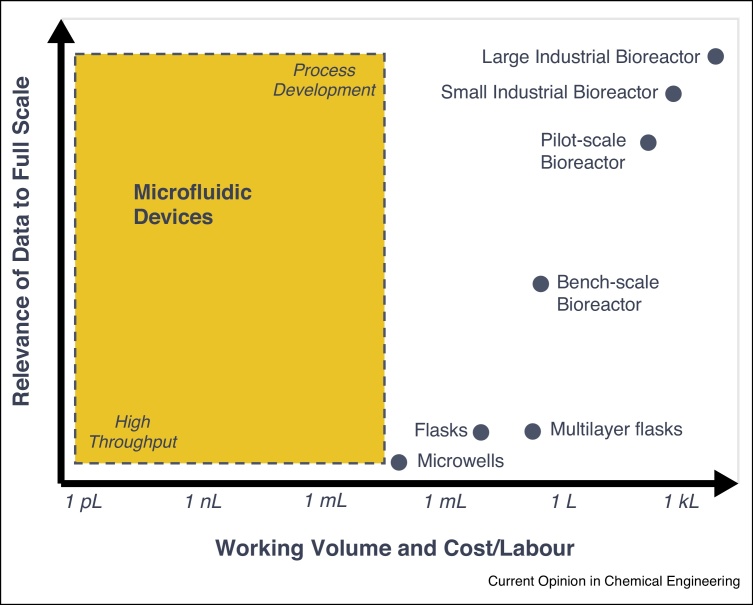
Microfluidic devices operating space for bioprocessing. A key challenge in bioprocessing is to obtain the desired information for process development with data that is relevant to the production scale in a cost-effective manner. Microfluidic devices with their precise control of the microenvironment can enhance the quality of the data while offering increased throughput. Furthermore, by combining them with sensing technology, these devices can be made suitable for process development.

A key challenge in bioprocessing is to gain an in-depth understanding of the bioprocesses to enable their rapid and successful development and implementation. This requires obtaining data that is relevant for the production scale with minimum amount of labor and at minimum cost. In addition to process variables (e.g. pH, oxygen, cell density), physiological and metabolic data, and also data regarding productivities need to be acquired [1]. At the production scale, all process information is available but the associated cost to perform process development is prohibitive. It is estimated that over 10 000 experiments are necessary for a single bioprocess development project, from primary strain screening to pilot-scale trials [2]. The key experiments are typically performed with bench-scale bioreactors. Though resulting in reliable and information rich data, this is still an expensive and labor intensive endeavor, and the number of experiments that can be carried out simultaneously are therefore constrained (Figure 1).

To reduce further cost and time involved in process development, miniaturized bioreactors are used as an alternative to bench-scale bioreactors. Miniaturized bioreactors, with volumes typically below 100 mL, comprise both stirred tank reactors and shaken systems, for example, shaken flasks and microtiter plates. Microtiter plates offer a high degree of parallelization, simplicity and operate with very small volumes. Furthermore, they enable automation of the optimization processes with commercially available laboratory robotic platforms minimizing time-consuming manual work while increasing throughput. When combined with design of experiments (DoE) approaches, the total number of experiments required is reduced which can minimize the number of more expensive larger scale experiments. Whilst the influence of operational and biochemical parameters on process yields can be obtained, the significance of the data obtained for larger scale is limited. For example, with shaken systems similar oxygen mass transfer characteristics can be obtained, however, hydrodynamics, transport phenomena and power input [3] differ significantly from pilot scale reactors. Furthermore, the data obtained per experiment can be limited due to a lack of process control and sensor integration. Despite efforts in the last decade to circumvent this by integrating sensing technology to monitor and control process variables, monitoring in these systems is still mostly limited to pH and dissolved oxygen (DO) [4].

Systems are sought which have the same level of throughput as shaken systems but are able to generate data with higher quality and preferably lower cost, in order to address this bioprocess development challenge. Microfluidic devices, with characteristic dimensions from submillimeter to submicrometers, and in which small amounts of fluids (10^−9^ and 10^−18^ l) are manipulated [5] have emerged as enabling tools for high throughput applications. As with traditional bench-scale reactors, they can in principle be operated in batch, fed-batch, and continuous modes under a broad range of process conditions. The flow regime imposed by the small dimensions is laminar, which gives fine control over the temporal and spatial microenvironment, ideal for the controlled and local delivery of reagents, cells or enzymes. With the introduction of novel fabrication technologies, different materials, the capability to modify surface conditions, and nano-structured topographies devices can be tailored to desired specifications. Monitoring of key process variables in microfluidic devices is typically achieved with optical sensors and similar luminescence quenching principles (e.g. to monitor oxygen, pH, CO_2_, glucose and temperature), by spectroscopy methods (e.g. NIR and Raman) [6•, 7•] and by image analysis [8]. Nonetheless, there is still a need to implement robust and sensitive online and in situ detection methods in order to obtain quality data and achieve a high level of process control.

In this review an overview on how microfluidic devices have been employed as tools for process development and what challenges still remain is presented. In particular, we will focus on how these devices can be applied to process development for the production of small molecules, therapeutic proteins, and cells, and discuss their functionalities in the context of these.

## Adherent cell culture devices

Microfluidic devices have been used in the past decade to study the cellular microenvironments of stem cells in a reproducible and controllable fashion. The fine spatio-temporal control over the cellular microenvironment enables controlled delivery of nutrients and other soluble biochemical factors, wash-out of auto and paracrine signaling factors, and the control over the forces exerted on the cells, such as the level of hydrodynamic shear stress. In conventional tissue culture dishes, this level of control is difficult if not impossible to achieve. Additionally, device parallelization offers increased throughput, and integration with analytical tools provides more relevant data per experiment [8, 9•, 10]. These advantages make microfluidic devices ideal to study cell responses, for example by applying small perturbations to the cellular microenvironment [11, 12, 13, 14, 15].

The incorporation of analytical tools in microfluidic cell culture devices (μCCD) is challenging due to the small dimensions and closed nature of the devices. Analytical methods should allow on-line monitoring to capture the dynamic behavior of the cells and be non-invasive, and options include light microscopy [16], optical sensors [17^••^] and electric cell-substrate impedance sensing (ECIS) [18]. Novel quantitative data can be obtained if these approaches are combined with automation, for example using image processing routines with live cell imaging [8, 10]. To further the understanding of cellular responses, these on-line methods can be complemented with off-line or at-line methods, such as FACS, HPLC and flow cytometry; either employing a sacrificial approach (i.e. harvesting the entire content of a culture chamber) or by coupling the effluent stream of the μCCD with the analytic equipment, respectively. Choosing the correct at-line or off-line analytical method and related protocol often represents a trade-off between the acceptable degree of disturbance of the cell culture and the information that can be obtained [5]. Non-invasive approaches avoid this trade-off, and real-time quantification of cellular responses, such as cell proliferation and oxygen kinetics (a key indicator of cell energy metabolism) has recently been successfully achieved from tiny amounts of cell culture medium and a small population of cells [9^•^].

Collecting a large amount of information with an integrated μCCD is of particular use when the production of cell and gene therapies are envisaged [19]. μCCDs only require a small starting cell population from patient samples, cells can be transfected with pluripotent factors [20] and maintained in continuous long-term cultures. The cells could then, after further expansion and differentiation, be transplanted back into the patient. No reports on cell reprogramming under perfusion conditions have been reported so far, despite advances in transfection workflows [21] and demonstrated advantages of perfusion cultures, such as increased proliferation rates [22] and better identification of reprogramming-enhancing extrinsic factors [23].

The spatiotemporal control over signaling gradients afforded by perfusion facilitates the transition from 2D to 3D cell cultures, obtaining better biomimetic tissues and organ models with increased physiological relevance [24, 25••, 26••]. These 3D systems can be used to test the adsorption, distribution, metabolism, elimination and toxicity (ADMET) of drugs, to support pharmacokinetics and pharmacodynamics modeling, to measure drug efficacy, thus ultimately for drug discovery [24, 27, 28]. For the bioindustry, this will mean tools which enable the validation and prioritization of drug candidates, and which could be used in pre-clinical or clinical trials to determine drug response, dosing and safety margins [29]. Such 3D systems may also facilitate comparison between organ models and results from clinical studies [25••, 26••].

To increase the uptake of μCCDs in industry, a few key technical challenges need to be addressed. Consistent and robust workflows of device sterilization and priming as well as cell seeding and culturing should be sought, in addition to the ability to monitor and manipulate cellular behavior. Standardization of μCCD and it components [30] will allow the implementation of Good Cell Culture Practices and integration with automated workflows enabled by liquid handling robots. Additionally, a basis for comparison of results between 2D cell monolayer models and 3D cultures and control mechanisms for 3D cultures are necessary to implement these system for cell therapies [26^••^].

## Flow biocatalysis — continuous enzymatic catalyzed reactions

Microfluidic devices have been widely applied in chemistry for organic synthesis [31, 32] due to a number of advantages, for example the enhanced mass transfer resulting from the high surface-to-volume ratios and short diffusion lengths. Whilst these advantages potentially apply to microfluidic devices for biocatalytic reactions, not all these advantages have yet been successfully demonstrated [33, 34•, 35•]; enhanced safety when handling potentially explosive compounds and point-of-use generation of toxic chemicals are not typical features of biocatalytic processes. In processes where enzymes are used as catalyst, reaction conditions are usually mild (e.g. temperatures lower than 100 °C and use of aqueous reaction medium) with no generation of toxic or explosive compounds. Nonetheless, microfluidic devices have been applied successfully in relevant reaction systems [34•, 35•] supporting the validity of a microfluidic approach for biocatalysis. These devices offer the possibility to rapidly and in a high throughput manner evaluate different reaction conditions and different enzyme variants, improve reaction stability, enable continuous processing [34^•^] and, in multi-phase systems, intensify processes [36].

Continuous processing offers an overall reduction of operation costs (e.g. by reducing the size of equipment, lowering energy consumption and reduced waste production) compared to batch operation mode. Additionally, product quality standards can be maintained along the entire operation time, facilitating repetitive or routine steps and enabling multistep syntheses [34^•^]. Multistep syntheses, such as cascades of chemo-enzymatic or enzyme–enzyme reactions, can benefit from microfluidic reactors [35^•^]: the spatial confinement of reactions into separate microreactors allows each reaction to be performed under the best possible conditions (as opposed to one-pot reactions where compromises between the individual reactions must be sought). In chemo-enzymatic reactions, where chemical reactions are proceeded by an enzymatic one (or vice versa), the major challenge lies in matching the reaction media to avoid enzyme inhibition or deactivation. Conversion yields can potentially be increased with the optimization of the chemical reaction step or with the aid of reactor engineering [35^•^], including the integration of in situ product removal (ISPR) strategies [37]. For enzyme–enzyme reactions, the challenges are significantly different and include matching reaction conditions, preventing cross-inhibition and overcoming enzyme inhibitions caused by reactants and products [38]. Despite the increasing number of coupled enzymatic reactions for the production of industrial relevant products the number of reports on the synthesis of organic compounds is still limited [35^•^]. Nonetheless, the modular approach to couple enzymatic reactions will pave the way to perform *in vitro* biosynthetic reactions in flow. New molecules can be produced by the creation of de novo pathways or cascades, which occur naturally through metabolic reactions in both cells and organs.

From an operational point of view, costs can be reduced and biocatalyst productivity (kg_product_ kg_biocatalyst_^−1^) increased with the recirculation or immobilization of the enzyme in the reactors. This will additionally improve enzyme stability and avoiding unwanted side reactions in cascade reactors. The choice between the use of free or immobilized enzymes should be assessed case-by-case and will depend on the activity and stability of the enzymes, operational constraints and overall cost and performance analysis [39]. Immobilization is typically carried out with the use of different surface geometries and chemistries. However, backpressure issues and complex liquid flow patterns in packed bed reactors and low volumetric productivity in wall-coated microreactors [40] should be taken into account and can limit the applicability of these reactors. Where free enzymes are the preferred option, these can be recycled using an in-line filtration step [35^•^].

The data obtained can be enhanced with the integration of sensor technology. This will provide real-time information on reaction progression [41•, 42], including reaction conditions (e.g. pH, temperature and oxygen), reaction parameters (e.g. substrate and products concentrations) and operational conditions (e.g. flow rates and pressure).

## Microbioreactors for submerged microbiological cultivations

Microbioreactors (μBR), bioreactors with working volumes in the submillitre range [6^•^], started with the seminal work of Kostov *et al.* [43^•^] with the integration of optical sensors in a stirred and sparged cuvette to monitor *Escherichia coli* fermentations, and were shortly after followed by the first microfluidic bioreactor with a culture chamber of 5 μl [6•, 7•]. Since then, a large number of μBRs have been developed and their functionality and instrumentation extended to render them suitable for process condition screening and process development [6•, 44•]. These developments were enabled by advances in polymer microfabrication and sensor miniaturization [17^••^]. Despite the lower throughput compared with microtiter plates (Figure 1), these reactors offer a controlled microenvironment and batch, fed-batch and continuous culture operation.

The majority of μBRs make use of the advantages of polymers, such as the exquisite gas permeability of polydimethylsiloxane (PDMS) for the aeration of the culture, or the relative ease of structuring polymers like poly(methyl methacrylate) (PMMA) or PDMS [45] to create fluidic structures. Additionally, they enable the fabrication of disposable devices either as a monolith or in a modular assembly [30]. The high optical transparency of the polymers facilitates integration of optical detection methods [17^••^]. The layout of most μBRs is planar and horizontal with mixing occurring in plane either by spin bars [6•, 7•] or by peristaltic motion of the chamber ceiling [46]. Recently, the group of Krull presented a vertically orientated bubble-column microreactor [47••, 48••] where the air bubbles not only contribute to the mixing effect but also to the overall volumetric mass transfer coefficient, reaching approximately 0.14 s^−1^.

Monitoring in μBRs is almost exclusively achieved by optical sensors due to their small footprint which allows easy integration and because they offer non-destructive measurement in tiny volumes, that is, without interfering cellular functions or the biological systems. These sensor have been shown to measure time profiles of pH, O_2_ and CO_2_, monitoring the individual analyte as well as multi-analyte monitoring [17^••^], and can be parallelized. More recently, specific oxygen uptake rate (sOUR) were measured in real time as an indicator of cell behavior and metabolism, both in planar as well as vertical μBR configurations [46, 48••].

To increase industrial uptake, however key technical challenges need to be addressed. Further developments in sensing technology are required to detect nutrient consumption and product formation. This will also allow the integration of mathematical models [44^•^] which will underpin Quality by Design (QbD) approaches for bioprocess development. Online monitoring of nutrients and product can be accomplished with fluorescence-based sensors, biosensors as well as potentially surface plasmon resonance and spectroscopic methods, for example, Raman; provided that the analytes exhibit Raman-activity and do not have overlapping Raman spectra. However, these technologies can be difficult to integrate in multi-analyte systems (e.g. cross signal interference) or will consume the analyte (e.g. glucose sensors). Additionally, conventional off-line or standard at-line analytical equipment (e.g., FACS, HPLC and flow cytometry) can be employed on a sacrificial based approach or by sampling ports, respectively. However, sampling is not trivial to implement due to low μBR working volumes (concomitantly reducing the working volume and altering process conditions) and by the increased risk of contamination. Standardization of device components is desired if automated workflows and process integration is considered. Furthermore, surface coating is necessary with current polymer based devices to avoid the adsorption of media components, must be sought to accomplish this endeavor. With standardization of device components, the integration of analytical solutions will be facilitated, in particular for chromatography or mass spectrometry [44^•^].

## Opportunities

**Figure 2:**
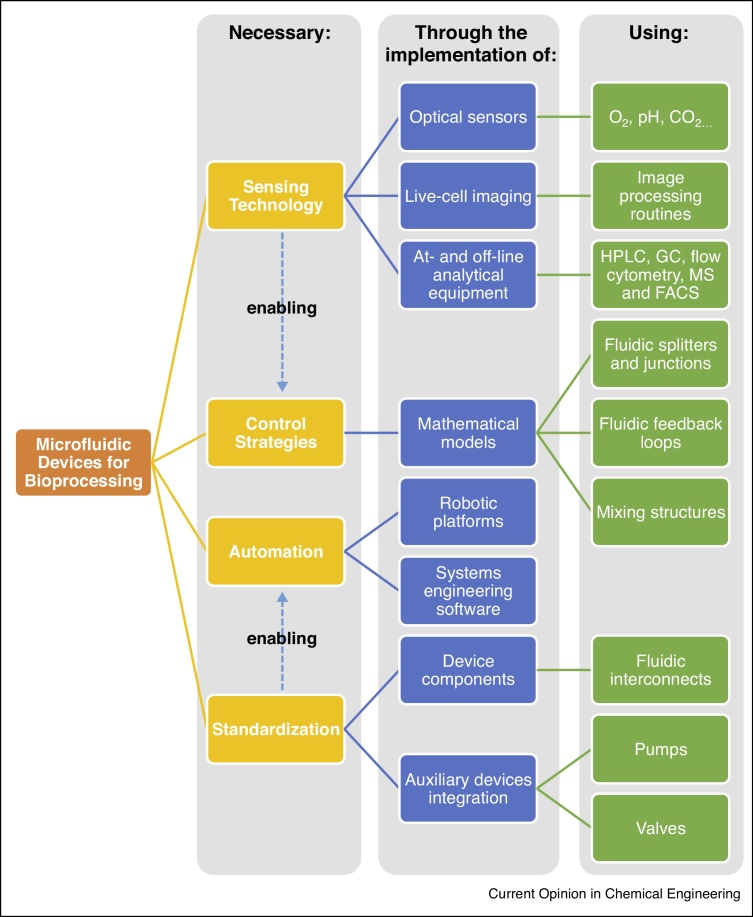
Enabling technologies for the implementation of microfluidic devices in bioprocessing. Efficient operation of microfluidic devices requires both the implementation of sensing technology, in the form of soft sensors, live-cell imaging or traditional analytical systems provided on-line measurements are supported. The fine and tight control over the cells microenvironment and process variables can be combined with various control strategies, such as, mathematical models. Standardization of device components and integration of auxiliary devices such as pumps and valves will facilitate the implementation of automation by the use of robotic platforms or in self-sustained automated platforms, and facilitate industrial uptake.

Since their inception, microfluidic devices for bioprocessing have matured significantly, though microbioreactors for submerged microbial cultivations and microreactors for biocatalysis are more developed than adherent cell culture devices (in the context of bioprocessing). This is partially also a reflection on the less developed scale-up trains for cellular therapies. The limitation for further uptake in industry of miniaturized devices lies in the lack of a more comprehensive monitoring of all process variables and sufficient automation, insufficient sample volume for quality control and integration of downstream processing. Device standardization and the development of robust and sensitive sensor technology are important for the implementation of Process Analytical Technology (PAT). Device standardization will also reduce the dependency of end-users on individual device manufacturers (or manufacturer of fluidic interconnects), which constitutes a significant economic risk for end-users to implement microfluidic technology in their workflow. Automation has the potential to remove operator-induced variability thus improving product quality consistency, increasing throughput and data quality. This can further be extended if microfluidic devices are integrated with robotic platforms. Equally important is the possibility to multiplex devices and operations with the ability to vary individual and control different process parameters (Figure 2).

There are opportunities for the uptake of microfluidic devices in downstream processing (μDSP) provided that we can properly exploit the design advantages of the microfluidic devices and combine these with sensor technology, which will deliver scale-relevant or scale-translatable data. μDSP have been far less developed, partially related to the difficulty to miniaturize traditional DSP system (e.g. due to complex geometries of the industrial scale equipment) [49^••^]. Nonetheless, there have been efforts to miniaturize DSP unit operations to the mL scale, coined an ‘ultra scale-down’ approach, where new units are designed maintaining equivalent process criteria (e.g. shear) to the larger scale unit [49^••^]. The data obtained at the small scale in conjunction with operational models will facilitate scale-translation of downstream processing units [49^••^].

In recent years, μDSP for process analysis have been reported, in solid–liquid based separations, either by adsorptive techniques [50, 51••] or use of membranes [52], liquid–liquid separations [53] and crystallization [54]. The applicability of these devices as process development tools depends greatly on the integration of analytics and the amount of samples available for quality control purposes. The integration of μDSP with upstream devices will enable the study of whole bioprocess sequences, having a significant impact in reducing development time and costs of full-scale processes. This approach will become increasingly of value when complex therapies require processing which are different than typical platform processes and rapid and cost-effective process development is necessary [55]. They could also potentially play a role for more stratified and more personalized therapies.

## References and recommended reading

Papers of particular interest, published within the period of review, have been highlighted as:

• of special interest
•• of outstanding interest
